# Precise placement of a triple-cavity drainage tube by guide wire exchange method for esophagojejunal anastomotic fistula after gastrectomy

**DOI:** 10.1186/s12957-023-03224-1

**Published:** 2023-10-27

**Authors:** Yuning Cao, Xiangheng Kong, Daogui Yang, Jianjun Li

**Affiliations:** 1 Department of Digestion, Liaocheng People’s Hospital, Liaocheng, Shandong Province China; 2 Department of Gastrointestinal Surgery, Liaocheng People’s Hospital, Liaocheng, 252000 Shandong Province China

**Keywords:** Esophagojejunal anastomotic fistula, Guide wire exchange method, Continuous irrigation and negative pressure suction, Triple-cavity drainage tube

## Abstract

This is a letter to the editor on a study by Ding et al. on the role of the three-tube method via precise interventional placement for esophagojejunal anastomotic fistula after gastrectomy. They suggest using transnasal insertion of abscess drainage catheter, jejunal decompression tube, and jejunal nutrition tube under fluoroscopy as a simple, minimally invasive, effective, and safe method for treating esophagojejunal anastomotic fistula. Compared to Ding et al.’s method, we presented a new procedure for the esophagojejunal anastomotic fistula. In this procedure, we precisely place a homemade triple-cavity drainage tube by guide wire exchange method near the esophagojejunal anastomotic fistula for continuous irrigation and negative pressure suction, which can provide adequate drainage and result in fistula’s self-healing. This procedure can also be performed at bedside without any anesthesia; therefore, it is a more simple, minimally invasive, effective, and safe treatment for esophagojejunal anastomotic fistula.

Dear Editor,

We read with interest the study by Ding et al. [1] in the August edition of the *World Journal of Surgical Oncology*. They suggest using insertion of transnasal abscess drainage catheter, jejunal decompression tube, and jejunal nutrition tube under fluoroscopy as a simple, minimally invasive, effective, and safe method for treating esophagojejunal anastomotic fistula after gastrectomy. We congratulate the authors Ding et al. on their retrospective study on the role of the three-tube method via precise interventional placement for esophagojejunal anastomotic fistula and its effect on patients’ survival.

Although the treatments of intestinal fistula include adequate drainage, nutritional support, and antibiotic therapy [2, 3], adequate drainage of intestinal contents out of the abdomen should be the most crucial part, because the main challenge of the intestinal fistula manifests in intraperitoneal leaks with consistent output and corrosive secretions [4]. Furthermore, adequate drainage can create a stable fistula tract that may spontaneously closed along with the drainage tube gradually pulled out, which then result in fistula’s self-healing.

With the aim of adequate drainage of esophagojejunal anastomotic fistula, continuous irrigation, and negative pressure suction by a homemade triple-cavity drainage tube consisted of an outer sleeve, a suction tube and an irrigation tube (Fig. 1), as demonstrated with effectivity in our previous published study [5], should be carried out.

**Fig. 1:**
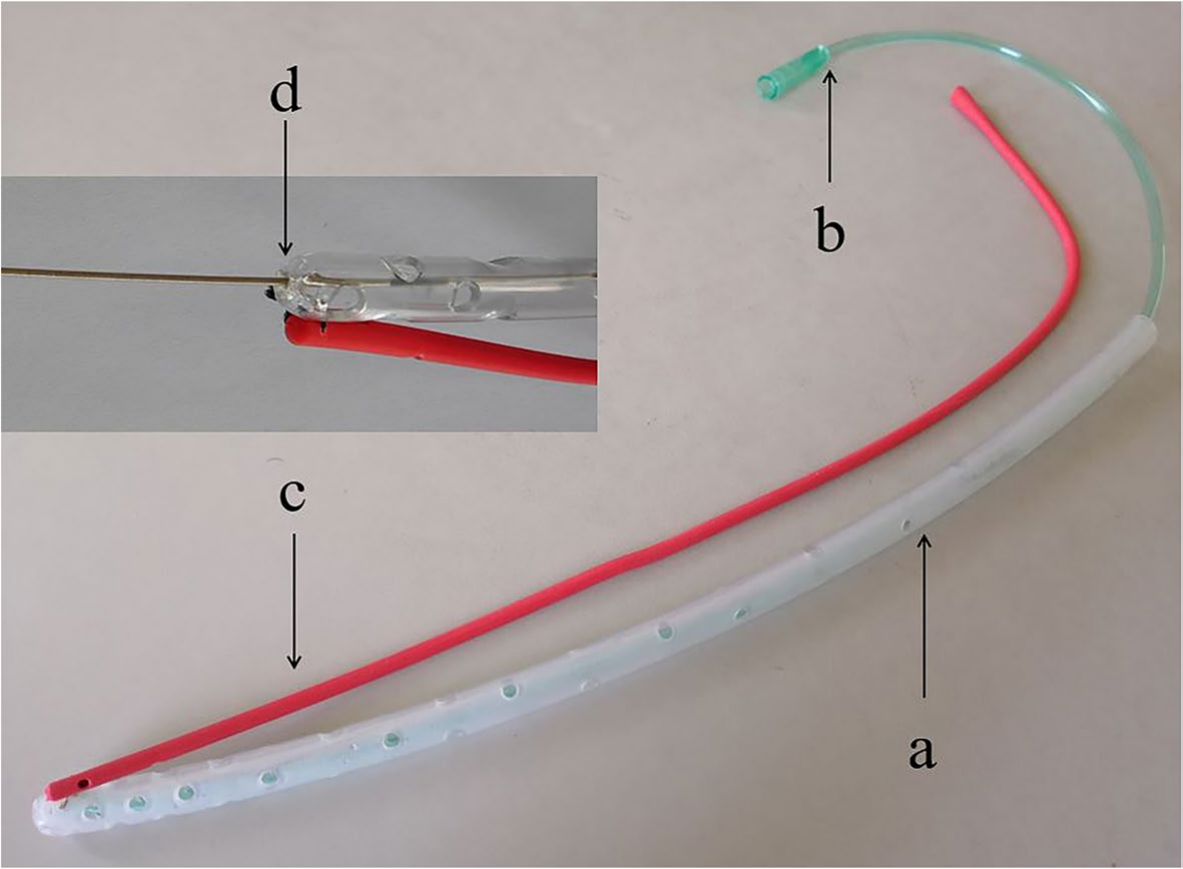
The homemade triple-cavity unit consisted of three different tubes: a large-bore silicone tube as an outer sleeve (**a**) with multiple side holes and two fine-bore tubes, including one for suction (**b**) inside the outer sleeve and another fixed to the outer sleeve for irrigation (**c**). The outer sleeve has a round top with a tiny hole for guide wire going through (**d**)

In our successful experiences, the treatment strategy for esophagojejunal anastomotic fistula involves properly intraoperative placement of abdominal drainage tube, jejunal decompression tube, and jejunal tube, as well as timely postoperative replacement of the homemade triple-cavity drainage tube when fistula occurs. As for the former, the abdominal drainage tube is always percutaneously placed through right inferior margin of costal arch, along superior margin of pancreas, and across below the esophagojejunal anastomosis (Fig. 2). Once fistula occurs, precise replacement of the abdominal drainage tube for the triple-cavity drainage tube should be immediately performed. However, it is difficult to accomplish this operation because the previous abdominal drainage tract is always too long and curved for the triple-cavity tube to pass through (Fig. 3). To solve this problem, we have devised a guide wire exchange method that has not been reported in any literatures.

**Fig. 2:**
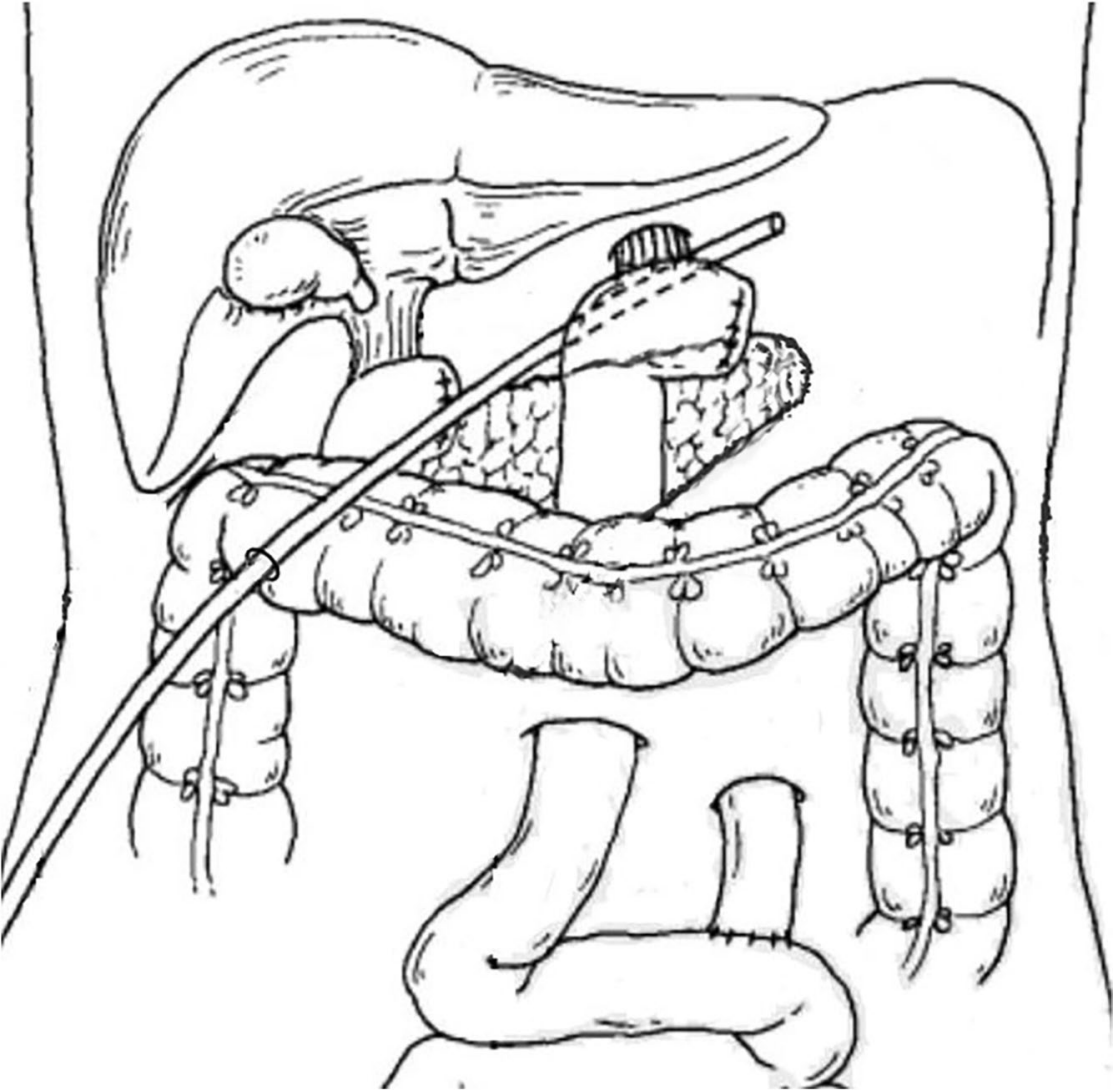
Diagram showed that abdominal drainage tube is placed along superior margin of pancreas and across below the esophagojejunal anastomosis

**Fig. 3:**
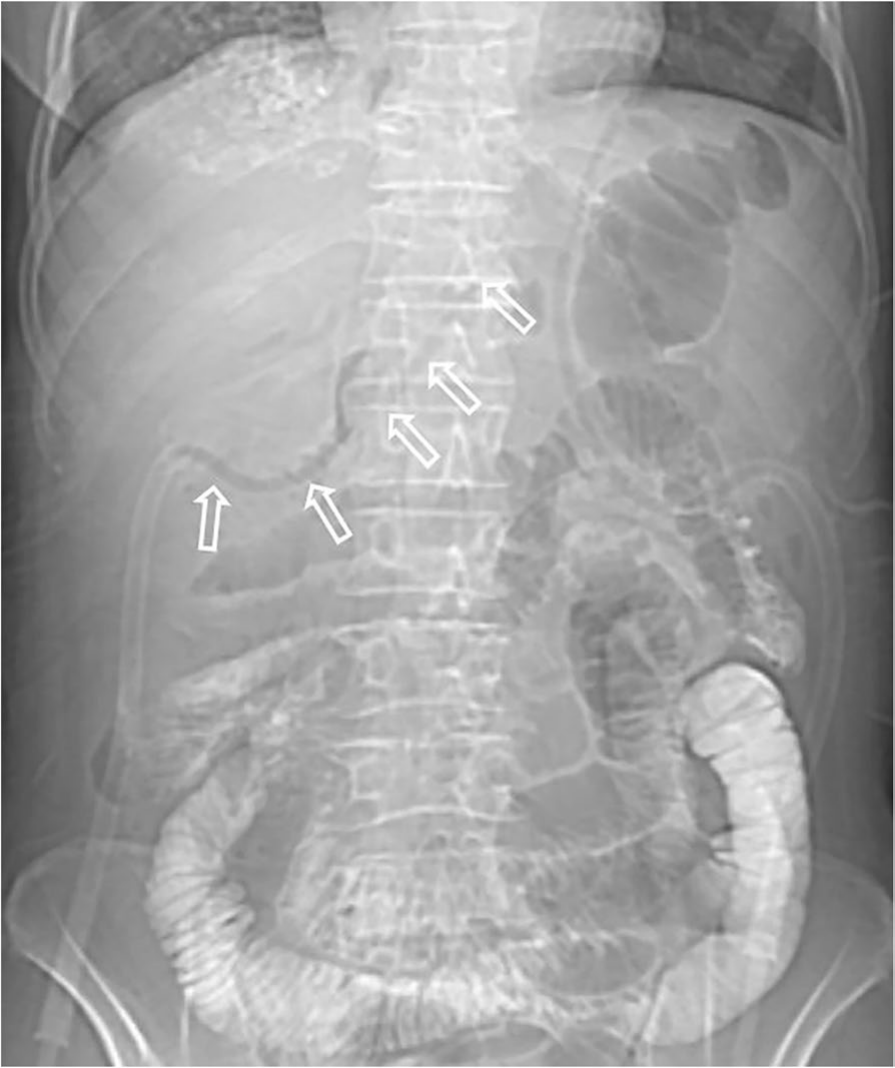
Computed tomography showed that the previous abdominal drainage tube is long and curved, which is difficult to be exchanged for triple-cavity tube

In this procedure, the guide wire is inserted through the previous abdominal drainage tube to the end. Then, the abdominal drainage tube was removed with the guide wire left in place. Finally, the triple-cavity drainage tube is advanced over the guide wire to get near the esophagojejunal anastomosis. After this procedure, computed tomography or fistulography with meglumine diatrizoate is performed to confirm the tube’s position (Fig. 4). Subsequently, the suction tube is connected to a negative-pressure (80–100 mmHg) suction system, and the irrigation tube is connected to saline.

**Fig. 4:**
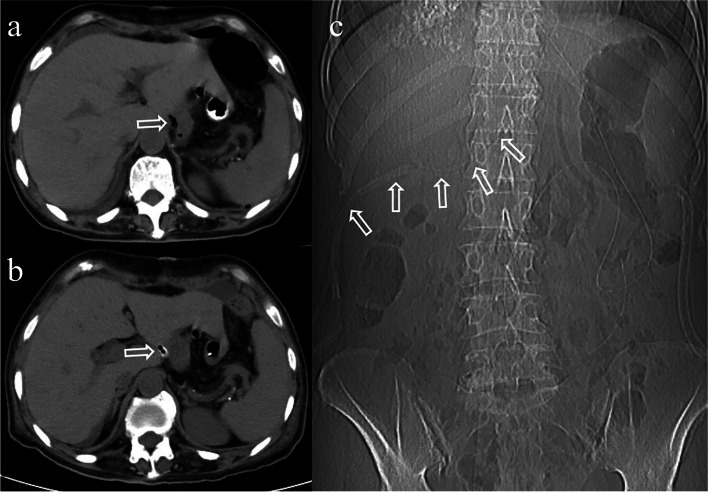
Computed tomography is performed to confirm the tube’s position. Photograph (**a**) showed the front end of the surgical drainage tube under the esophagojejunal anastomosis. **b** Showed the triple-cavity tube takes the place of the surgical drainage tube under the esophagojejunal anastomosis, which is precise placed by guide wire exchange method. **c** Showed the path of the triple-cavity tube

Compared to Ding’s method, our procedure can not only provide accurate and adequate drainage but also be performed at bedside without any anesthesia. In addition, placement of jejunal decompression tube and jejunal nutrition tube is performed intraoperatively, which avoid the second fluoroscopy operation.

Although thirteen of fifteen consecutive cases of esophagojejunal anastomotic fistulas in recent 5 years have been successfully treated by this procedure, it has a limitation that the guide wire must be inserted through the abdominal drainage tube. If the fistula occurs after the previous drainage tube removed, the guide wire can be not placed, which lead to a great difficulty in precise placement of the triple-cavity drainage tube.

In conclusion, precise placement of a triple-cavity drainage tube by the guide wire exchange method is an alternative, simple, minimally invasive, effective, and safe procedure for treating esophagojejunal anastomotic fistula after gastrectomy.

## Data Availability

All data generated or analyzed during this study are included in this published article.
